# Madura foot: a case report

**DOI:** 10.11604/pamj.2018.30.131.15045

**Published:** 2018-06-14

**Authors:** Ahmed Bouhamidi, Mohammed Boui

**Affiliations:** 1 Military Hospital of Instruction Mohammed V, Department of Dermatology, Rabat, Morocco

**Keywords:** Madura foot, black grains, eumycetoma

## Image in medicine

A 24-year-old girl, without any significant medical history. with seven months' history of a painful tumefaction of her left foot. Physical examination revealed a tumefaction that discharges a purulent exudate containing black granules via skin fistulas. Direct microscopic examination of grains suggests a fungal infection. Standard X-rays of his foot showed no lesion of the bone. So with the combination of the clinical specific lesions and typical grains, a diagnosis of Madura foot was made and the patient treated with surgical debridement, followed by Ketoconazole for 12 months, she is under regular monitoring. Mycetoma is characterized by a clinical triad of chronic induration, draining sinuses and discharge of granules. It is either actinomycotic or eumycotic in etiology. It is endemic in tropical and subtropical countries.

**Figure 1:**
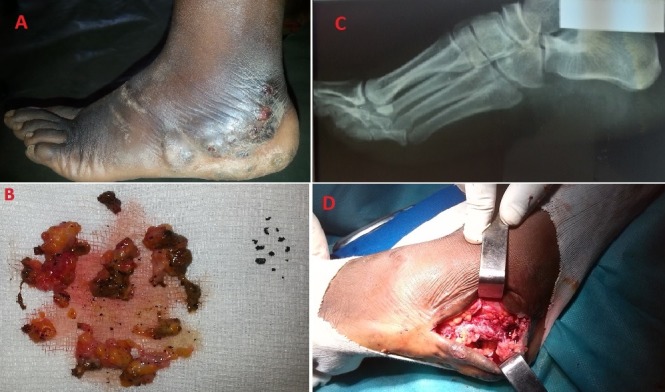
(A) tumefaction of the left foot discharging black grains; (B) black eumycetoma grains; (C) standard X-rays showed no bone lesion; (D) surgical debridement

